# Vitamin D Deficiency Aggravates Nephrotoxicity, Hypertension and Dyslipidemia Caused by Tenofovir: Role of Oxidative Stress and Renin-Angiotensin System

**DOI:** 10.1371/journal.pone.0103055

**Published:** 2014-07-21

**Authors:** Daniele Canale, Ana Carolina de Bragança, Janaína Garcia Gonçalves, Maria Heloisa Massola Shimizu, Talita Rojas Sanches, Lúcia Andrade, Rildo Aparecido Volpini, Antonio Carlos Seguro

**Affiliations:** Nephrology Department, University of São Paulo School of Medicine, São Paulo, Brazil; Federal University of São Paulo (UNIFESP), Escola Paulista de Medicina, Brazil

## Abstract

Vitamin D deficiency (VDD) is prevalent among HIV-infected individuals. Vitamin D has been associated with renal and cardiovascular diseases because of its effects on oxidative stress, lipid metabolism and renin-angiotensin-aldosterone system (RAAS). Tenofovir disoproxil fumarate (TDF), a widely used component of antiretroviral regimens for HIV treatment, can induce renal injury. The aim of this study was to investigate the effects of VDD on TDF-induced nephrotoxicity. Wistar rats were divided into four groups: control, receiving a standard diet for 60 days; VDD, receiving a vitamin D-free diet for 60 days; TDF, receiving a standard diet for 60 days with the addition of TDF (50 mg/kg food) for the last 30 days; and VDD+TDF receiving a vitamin D-free diet for 60 days with the addition of TDF for the last 30 days. TDF led to impaired renal function, hyperphosphaturia, hypophosphatemia, hypertension and increased renal vascular resistance due to downregulation of the sodium-phosphorus cotransporter and upregulation of angiotensin II and AT1 receptor. TDF also increased oxidative stress, as evidenced by higher TBARS and lower GSH levels, and induced dyslipidemia. Association of TDF and VDD aggravated renovascular effects and TDF-induced nephrotoxicity due to changes in the redox state and involvement of RAAS.

## Introduction

Tenofovir disoproxil fumarate (TDF) is a nucleotide reverse transcriptase inhibitor commonly used for treatment of HIV infection and hepatitis B on the basis of its effectiveness in clinical trials [1]–[4]. However, the long-term use of TDF has been associated with hypophosphatemia due to proximal renal tubular dysfunction, renal failure [5] and enhancement of oxidative stress by disruption of mitochondrial DNA in proximal tubule cells [6]. In addition to the renal impairment caused by TDF-induced nephrotoxicity it has been recently shown that low vitamin D levels are associated with the progression of HIV-related diseases in patients receiving antiretroviral therapy [7], [8].

Vitamin D is an indispensable nutrient for mineral homeostasis [9] and is also responsible for kidney protection and the regulation of several renal physiological activities [10]. Thus, vitamin D deficiency (VDD) (<10 ng/mL) or insufficiency (10–30 ng/mL) can accelerate the progression of kidney disease [10]–[13].

Vitamin D has been associated with renal and cardiovascular diseases because of its effects on oxidative stress, lipid metabolism and renin-angiotensin-aldosterone system (RAAS). *Tarcin et al* demonstrated that vitamin D deficient individuals presented higher plasma levels of Thiobarbituric Acid Reactive Substances (TBARS) [14]. Besides the effects of VDD on oxidative stress, studies have demonstrated that HIV-infected individuals exhibit a deficiency of total glutathione (GSH) [15] aggravating the redox state. Furthermore, several studies have shown that vitamin D is a negative endocrine regulator of RAAS [16] and its concentration has been inversely associated with the prevalence of metabolic syndrome [17].

Given that VDD is highly prevalent among HIV-infected individuals, the aim of this study was to investigate the effects of VDD on TDF-induced nephrotoxicity, mainly focused on the role of oxidative stress and RAAS.

## Materials and Methods

All experimental procedures were approved by the local Research Ethics Committee (CEP-FMUSP – Comitê de Ética em Pesquisa da Faculdade de Medicina da Universidade de São Paulo), protocol number 086/11). All experiments were developed in strict conformity with local institutional guidelines and with well-established international standards for manipulation and care of laboratory animals.

### Animals and experimental protocol

Male Wistar rats, weighing 200–250 g, were obtained from the animal facilities of the University of São Paulo School of Medicine, housed in standard cages, and given *ad libitum* access to water and commercial rodent chow, standard (Cat.# 960397) or vitamin D-free (Cat.# 960074), both obtained from *MP Biomedicals* (MP Biomedicals, Irvine, CA, USA). Rats were randomly allocated to the following groups: control (C, n = 10), receiving a standard diet for 60 days; VDD (n = 7), receiving a vitamin D-free diet for 60 days; TDF (n = 10), receiving a standard diet for 60 days with the addition of TDF (50 mg/kg food) for the last 30 days; and VDD+TDF (n = 8) receiving a vitamin D-free diet for 60 days with the addition of TDF (50 mg/kg food) for the last 30 days. The dose of TDF was based on a previous study from our laboratory [5] and is compatible with the dosage administered to humans.

### Metabolic cage studies and analysis of urine samples

At the end of the protocol, rats were moved to metabolic cages (one rat per cage), maintained on a 12-h light/dark cycle and given free access to drinking water. The rats were acclimated to the housing conditions for 1 day before the experimental procedures, which began with the collection of 24-h urine samples. The volume of each 24-h urine sample was measured gravimetrically. Urine samples were centrifuged in aliquots to remove suspended material, and the supernatants were analyzed. Urine concentrations of sodium and potassium were determined with specific electrodes (ABL800Flex - Radiometer, Brønshøj, Denmark). Urinary potassium/sodium ratio was calculated (U_K_/U_Na_). Urine concentrations of phosphorus, calcium and protein were measured by a colorimetric system using a commercial kit (Labtest Diagnóstica – Minas Gerais, Brazil). Urinary excretions of phosphorus (U_P_V), calcium (U_Ca_V) and protein (U_Prot_V) were determined.

### Hemodynamic studies

To determine glomerular filtration rate (GFR), inulin clearance studies were conducted at the end of the protocol. On the day of the experiment, the animals were anesthetized intraperitoneally with sodium pentobarbital (50 mg/kg BW). The trachea was cannulated with a PE-240 catheter, and spontaneous breathing was maintained. To control mean arterial pressure (MAP) and allow blood sampling, a PE-60 catheter was inserted into the right carotid artery. MAP was assessed using Biopac Systems Inc MP100 (Santa Barbara, CA, USA). For the infusion of inulin and fluids, another PE-60 catheter was inserted into the left jugular vein. In order to collect urine samples, a suprapubic incision was made, and the urinary bladder was cannulated with a PE-240 catheter. After the surgical procedure has been completed, a loading dose of inulin (100 mg/kg BW diluted in 0.9% saline) was administered through the jugular vein. Subsequently, a constant infusion of inulin (10 mg/kg BW in 0.9% saline) was started and kept at 0.04 ml/min throughout the experiment. Three urine samples were collected at 30-min intervals. Blood samples were obtained at the beginning and at the end of the experiment. Blood and urine inulin were determined using the anthrone method. GFR data are expressed as ml/min/100 g.

To measure renal blood flow (RBF), a midline incision was made. Then, we carefully dissected the left renal pedicle and isolated the renal artery, taking precautions to avoid disturbing the renal nerves. An ultrasonic flow probe was placed around the exposed renal artery. RBF was measured using an ultrasonic flowmeter (T402; Transonic Systems, Bethesda, MD, USA) and is expressed as ml/min. Renal vascular resistance (RVR) was calculated by dividing the blood pressure by RBF and is expressed as mmHg/ml/min.

### Evaluation of 25-hydroxyvitamin D [25(OH)D]

For assessment of plasma levels of circulating vitamin D [25(OH)D], we used a radioimmunoassay (RIA) commercial kit from DiaSorin (DiaSorin, MN, USA). Briefly, this method consists of two-step procedure: (1) a rapid extraction of 25(OH)D from plasma with acetonitrile; (2) following extraction, the treated sample is then assayed using an equilibrium RIA procedure, based on an antibody with specificity to 25(OH)D.

### Assessment of plasma biochemical parameters

For evaluation of PTH, aldosterona, sodium (P_Na_), potassium (P_K_), phosphate (P_P_), calcium (P_Ca_), cholesterol and triglycerides blood samples were collected. PTH was assessed by Enzyme-Linked Immunosorbent Assay (ELISA) using a commercial kit (Rat Bioactive Intact PTH (Immutopics - California, USA), aldosterone was determined by radioimmunoassay (RIA) using a commercial kit (Immunotech, Marseille, FR), P_P_ was evaluated by a colorimetric system using a commercial kit (Labtest Diagnóstica – Minas Gerais, Brazil) and P_Na_, P_K_ and P_Ca_ were determined with specific electrodes (ABL800Flex – Radiometer, Brønshøj, Denmark). Cholesterol and triglycerides were evaluated by a colorimetric system using a commercial kit (Cobas C111 - Roche, São Paulo, Brazil).

### Tissue sample collection/preparation

At the end of the experiments, the organs were perfused with PBS solution (0.15 M NaCl and 0.01 M sodium phosphate buffer, pH 7.4). The left kidney were removed and weighed. For histological/immunohistochemical examination, a fragment of the right kidney was fixed in 10% neutral-buffered formalin solution. The kidney block was dehydrated in graded alcohol, embedded in paraffin and cut at 4-µm sections. Another fragment of the right kidney was frozen in liquid nitrogen and stored at −80°C for assessment of endothelial nitric oxide syntase (eNOS), sodium-phosphate cotransporter subtype IIa (NaPi-IIa), AT1 receptor (AT1r) and angiotensinogen/angiotensin I and II (Ang) protein expression.

### Light microscopy studies

Four-µm histological sections of renal tissue were stained with hematoxylin-eosin and examined under light microscope. For the evaluation of renal damage, 40–60 grid fields (×400 magnification) measuring 0.245 mm^2^ were evaluated by graded scores according to the following criteria: (0), less than 5% of the field showing tubular epithelial swelling, vacuolar degeneration, necrosis, and desquamation; (I), 5–25% of the field presenting renal lesions; (II), involvement of 25–50% with renal damage; (III), 50–75% of damaged area; and (IV), more that 75% of the grid field presenting renal lesions. The morphometric examination was blinded to minimize observer bias, i.e. the observer was unaware of the treatment group from which the tissue originated. The mean score for each rat and the mean score for each group were calculated.

### Immunohistochemistry studies

Four-µm sections of kidney were incubated 60 min at room temperature (1/1,000) with a polyclonal primary antibody anti-angiotensin II (Santa Cruz Biotechnology). The reaction product was detected with an avidin-biotin-peroxidase complex (Vector Laboratories, Burlingame, CA). The color reaction was developed with 3,3-diaminobenzidine (Sigma), and the material was counterstained with Harris hematoxilin, dehydrated, and mounted.

### Preparation of samples for the determination of eNOS, NaPi-IIa, AT1r and Angiotensinogen (AGT) protein expression

In order to quantify eNOS, NaPi-IIa, AT1r and AGT protein expression, kidney sections were prepared. The sections were homogenized using a Teflon pestle glass homogenizer (Schmidt and Co.) in an ice-cold isolation solution of 200 mM mannitol, 80 mM HEPES and 41 mM KOH, pH 7.5, also containing a protease inhibitor cocktail (Sigma). The homogenates were centrifuged at a low speed (3000×g) for 15 min at 4°C to remove nuclei and cell debris. The pellets were suspended in an isolation solution with protease inhibitors. Protein quantities were determined using the Bradford assay method (Bio-Rad Protein Assay kit, Bio-Rad Laboratories, Hercules, CA, USA).

### Electrophoresis and immunoblotting

Kidney samples were run on 8% polyacrylamide minigels (for eNOS) or 10% polyacrylamide minigels (for NaPi-IIa, AT1r and Ang). After transfer by electroelution to nitrocellulose membranes (PolyScreen, PVDF Transfer, Life Science Products, Boston, MA, USA), blots were blocked with 5% skin milk and 0.1% Tween 20 in TBS for 1 hour. Blots were then incubated overnight with an anti-eNOS antibody (1∶2,000), anti-NaPi-IIa antibody (0.54 µg/mL), anti-AT1r antibody (1∶500) and anti-AGT antibody (1∶1,000). The labeling was visualized with a horseradish peroxidase-conjugated secondary antibody (anti-mouse IgG diluted 1∶2,000, anti-rabbit IgG, diluted 1∶2,000 or anti-goat IgG diluted 1∶10,000) using the enhanced chemiluminescence detection system ECL Western blotting detection reagents (GE Healthcare, Buckinghamshire, UK). The specific polyclonal antibody to NaPi-IIa was kindly supplied by Dr Mark Knepper (NHLBI/NIH, Bethesda, MD, USA). The specific polyclonal antibody to eNOS was obtained from BD Transduction Laboratories (CA, USA) and specific polyclonal antibody to AT1 and angiotensin II were obtained from Santa Cruz Biotechnology (CA, USA). As a loading control, blots were incubated with an actin antibody (Santa Cruz, CA, USA; 1∶2,000 with anti-goat 1∶10,000).

### Quantification of renal levels of eNOS, NaPi-IIa, AT1r and AGT

The images were obtained using chemiluminescence imaging system Alliance 4.2 (Uvitec, Cambridge, UK) and performed quantitative analysis of antibodies using densitometry, normalizing the bands to actin expression.

### Gene expression of renin-angiotensin components

Quantitative real-time PCR (qPCR) was performed in frozen renal tissue to analyze the expression of genes deemed likely to be related to the development of hypertension in rats. The following genes were assessed: renin (Rn00561847_m1), angiotensinogen (AGT – Rn00593114_m1), angiotensin converting enzyme (ACE – Rn00561094_m1) and angiotensin II receptor type a (AT1a – Rn02758772_s1). The extraction and preparation of total RNA were performed. For cDNA synthesis, total RNA and a Superscript VILO MasterMix (Invitrogen Technologies, CA, USA) were employed. Real-time PCR was performed using TaqMan (Applied Biosystem, CA, USA) on Step One Plus (Applied Biosystem, CA, USA). All primers were purchased from Invitrogen Technologies. Relative gene expression values were evaluated with the 2^−ΔΔCt^ method [18] using GAPDH as housekeeping gene.

### Reactive oxygen metabolites

Urinary and plasma levels of thiobarbituric acid reactive substances (TBARS), which are markers of lipid peroxidation, were determined using the thiobarbituric acid assay. In brief, a 0.2-ml sample was diluted in 0.8 ml of distilled water. Immediately thereafter, 1 ml of 17.5% trichloroacetic acid was added. Following the addition of 1 ml of 0.6% thiobarbituric acid, pH 2, the sample was placed in a boiling water bath for 15 min, after which it was allowed to cool. Subsequently, 1 ml of 70% trichloroacetic acid was added, and the mixture was incubated for 20 min. The sample was then centrifuged for 15 min at 2000 rpm. The optical density of the supernatant was read at 534 nm against a blank reagent using a spectrophotometer. The concentration of lipid peroxidation products was calculated as malondialdeide (MDA) equivalent using a molar extinction coefficient for the MDA-thiobarbituric acid complex of 1.56×105 mol^−1^/cm^−1^. Urinary and plasma levels of TBARS were expressed as nM/24 hours and nM/mL, respectively [19].

Renal reduced glutathione (GSH) was determined in total blood by the method of Sedlak and Lindsay [20]. Whole blood was processed by addition of four volumes of ice-cold 5% (W/V) metaphosphoric acid (MPA) (Sigma) and centrifuged at 14,000×g for 3 min. This assay consists of reacting the supernatants of the total blood with Ellman’s reagent to produce a yellow pigment measured spectrophotometrically at 412 nm. The GSH was quantified by mean of standard curve and reported as µmol of GSH/mL [21].

### Statistical analysis

All quantitative data are expressed as mean ± SEM. Differences among the means of multiple parameters were analyzed by one-way analysis of variance followed by the Student–Newman–Keuls test. Values of P<0.05 were considered statistically significant.

## Results

The animals were maintained on a standard or a free-vitamin D diet for 30 days. After this time, VDD animals had low levels of 25(OH)D (4.10±0.8 ng/mL) compared to respective control animals (14.79±0.9 ng/mL), demonstrating that these animals were already deficient before treatment with TDF.

As described in Table 1, there were no significant changes in body weight among groups since all animals showed similar food ingestion during 60 days (approximately 25 g/day). Rats treated with TDF presented a higher blood pressure and significantly impaired renal function, evidenced by lower inulin clearance compared to control group. These alterations were accompanied by a renal vasoconstriction. VDD animals showed a decreased inulin clearance compared to control rats, without alterations in blood pressure and renal vascular resistance. Treatment of VDD with TDF resulted in an even greater elevation of blood pressure than in the TDF-group associated with a greater decrease in inulin clearance and an intense vasoconstriction, indicating that vitamin D deficiency is an aggravating factor in the progression of TDF-induced renal injury.

**Table 1:** Body weight, renal function and hemodynamic measurements in normal (control) rats, in rats maintained 60 days on vitamin D free diet (VDD), in rats on normal diet treated for additional 30 days with tenofovir (TDF) and in rats on vitamin D free diet for 60 days, to which tenofovir was added on day 30 and continued throughout (VDD+TDF).

	C	VDD	TDF	VDD+TDF
BW	498±12	476±7	461±6	463±14
GFR	0.88±0.04	0.61±0.06^b^	0.63±0.04^c^	0.45±0.04^c,d^
MAP	117±3	127±4	134±2^b^	148±5^c,e^
RBF	5.58±0.03	5.82±0.04	5.59±0.05	5.72±0.08
RVR	19.8±0.4	21.1±0.8	24.1±0.5^c^	26.9±1.0^c,e^

BW, body weight (g); GFR, inulin clearance (mL/min/100 g); MAP, mean arterial pressure (mmHg); RBF, renal blood flow (mL/min); RVR, renal vascular resistance (mmHg/mL/min). Values are means ± SEM. ^a^p<0.05, ^b^p<0.01, ^c^p<0.001 vs. C; ^d^p<0.05; ^e^p<0.01 vs. TDF.

Another marker of renal impairment is proteinuria. As shown in Table 2, rats that received TDF, deficient or not in vitamin D, showed a greater loss of urinary protein compared to the control group. Urinary protein excretion was also increased in the VDD group. It is worth considering that if we had evaluated urinary fraction excretion of proteins of VDD+TDF group, it would be higher due to decreased glomerular filtration rate with subsequent lower protein filtered load.

**Table 2:** Biochemical parameters measurements in normal (control) rats, in rats maintained 60 days on vitamin D free diet (VDD), in rats on normal diet treated for additional 30 days with tenofovir (TDF) and in rats on vitamin D free diet for 60 days, to which tenofovir was added on day 30 and continued throughout (VDD+TDF).

	C	VDD	TDF	VDD+TDF
25(OH)D	15.4±1.0	<1.5 (undetectable)^c^	14.8±1.3	<1.5 (undetectable)^c,e^
PTH	120±11	1261±33^c^	83±15	516±151^b,e^
Aldosterone	116±15	243±46	316±57^a^	479±83^c,d^
P_P_	9.38±0.40	5.03±0.11^c^	6.47±0.34^c^	6.29±0.39^c^
P_Ca_	1.13±0.01	0.94±0.05^c^	1.14±0.02	0.94±0.03^c,f^
P_Na_	136±1	139±3	136±1	138±1
P_K_	3.9±0.3	4.3±0.2	3.7±0.2	4.1±0.2
Cholesterol	39±1	53±4^a^	54±3^a^	80±7^c,f^
Triglycerides	17±4	34±5	59±9^b^	74±13^c^
U_P_V	14.9±1.1	17.0±0.9	18.7±0.8^a^	21.5±0.7^c,d^
U_Ca_V	0.08±0.01	0.13±0.01	0.11±0.02	0.13±0.01
U_K_/U_Na_	3.27±0.17	3.39±0.16	3.07±0.18	4.46±0.49^a,e^
U_Prot_V	5.3±0.3	11.3±0.8^c^	10.8±0.3^c^	11.8±0.5^c^

25(OH)D, 25 hydroxyvitamin D (ng/mL); PTH, parathormone (pg/mL); Aldosterone (pg/mL); P_P_, plasma phosphate concentration (mg/dL); P_Ca_, plasma calcium concentration (mmol/L); P_Na_, plasma sodium concentration (mEq/L); P_K_, plasma potassium concentration (mEq/L); Cholesterol, total cholesterol (mg/dL); Triglycerides (mg/dL); U_P_V, urinary phosphorus excretion (mg/day); U_Ca_V, urinary calcium excretion (mg/day); U_K_/U_Na_, urinary concentration of potassium and sodium ratio; U_Prot_V, urinary protein excretion (mg/day). Values are means ± SEM. ^a^p<0.05, ^b^p<0.01, ^c^p<0.001 vs. C; ^d^p<0.05; ^e^p<0.01, ^f^p<0.001 vs. TDF.

**Figure 1:**
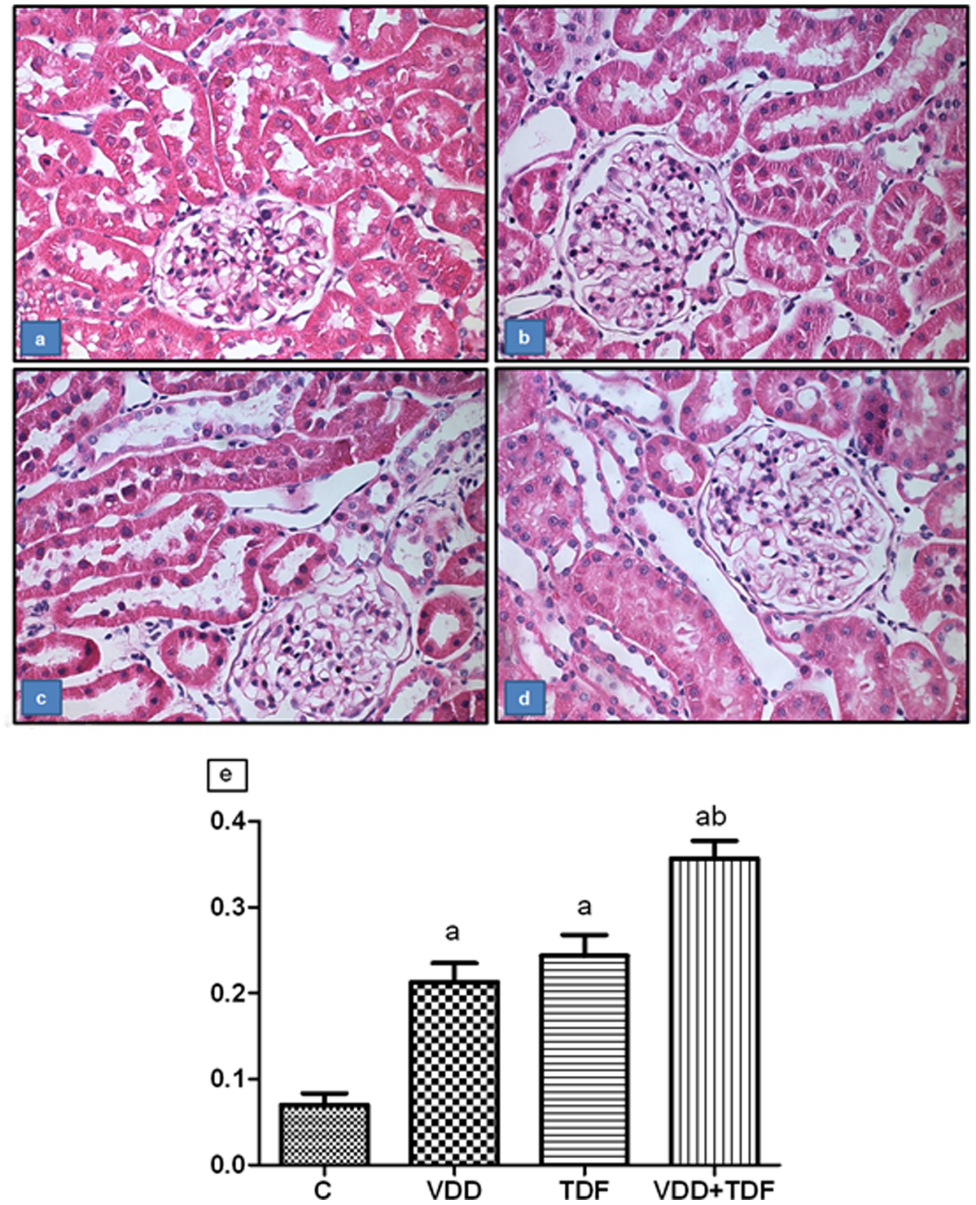
Representative photomicrographs of the tubular injury. (a) control, (b) VDD, (c) TDF and (d) VDD+TDF. Magnification, 400x. (e) Score of tubular injury of C, VDD, TDF and VDD+TDF. Values are means ± SEM. ^a^p<0.001 vs. C; ^b^p<0.001 vs. TDF.

The histological study showed alterations characteristic of mild acute tubular necrosis in the renal cortex from the VDD (0.21±0.02) and TDF (0.24±0.02) groups compared to control (0.07±0.01). Tubular cell necrosis, focal areas of denuded basement membrane, flattening of proximal tubular cells with brush border loss and tubular atrophy or dilatation were observed. Vitamin D deficient animals treated with TDF (0.36±0.02) exhibited a higher tubular injury score compared to groups VDD and TDF (Figure 1).

**Figure 2:**
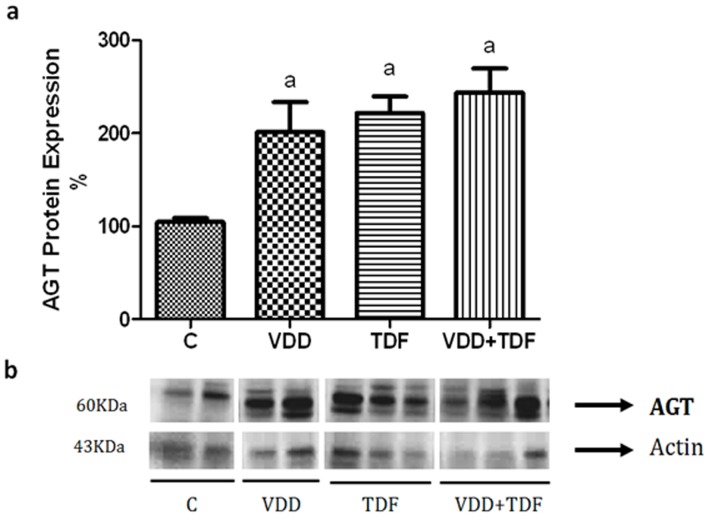
Semiquantitative immunoblotting of kidney fractions. (a) A densitometric analysis of samples from control (n = 4), VDD (n = 6), TDF (n = 6) and VDD+TDF (n = 6) rats is shown. (b) Immunoblots reacted with anti-angiotensinogen revealing a 60-kDa band. Values are means ± SEM. ^a^p<0.05 vs. C.

**Figure 3:**
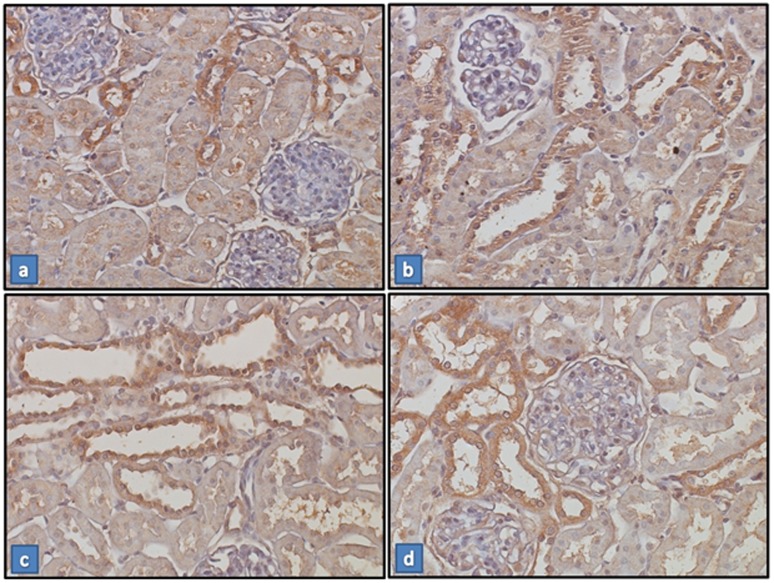
Expression of Angiotensin II in renal tissue. Immunostaining for angiotensin II (brown) in kidney cortex samples from C (a), VDD (b), TDF (c) and VDD+TDF (d). Magnification, ×400. Note that the staining is more extensive and intense in the sample from the VDD, TDF and VDD+TDF.

**Figure 4:**
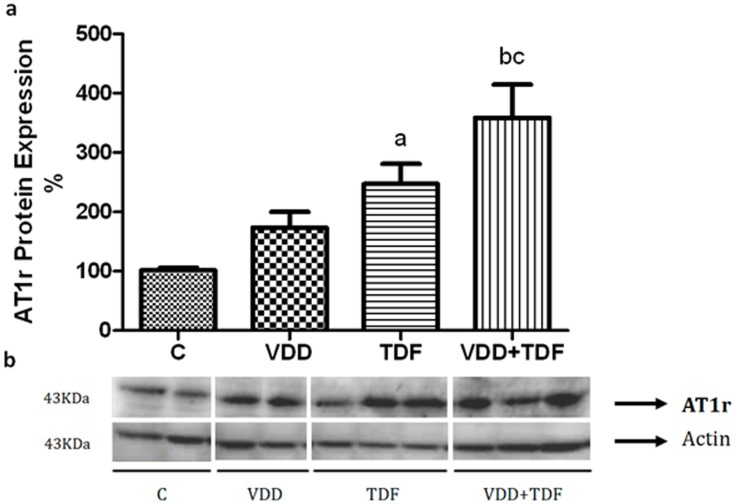
Semiquantitative immunoblotting of kidney fractions. (a) A densitometric analysis of samples from control (n = 4), VDD (n = 6), TDF (n = 6) and VDD+TDF (n = 6) rats is shown. (b) Immunoblots reacted with anti-AT1 revealing a 43-kDa band. Values are means ± SEM. ^a^p<0.05 vs. C; ^b^p<0.01 vs. C and ^c^p<0.05 vs. TDF.

Renal protein expression of angiotensinogen (Figure 2) was up-regulated in groups VDD (201±32%), TDF (222±18%) and VDD+TDF (244±26%) compared to control (104±45%). The immunohistochemical study showed an increased intensity of the immunoreaction of angiotensin II in the renal cortex of VDD, TDF and VDD+TDF (Figure 3). To support data previously shown, renal protein expression of AT1 receptor (Figure 4) was also up-regulated in TDF rats (247±33%) compared to control and VDD (101±5% and 173±27%). Treatment of VDD with TDF resulted in a more intense up-regulation of AT1 protein expression (359±56%).

**Figure 5:**
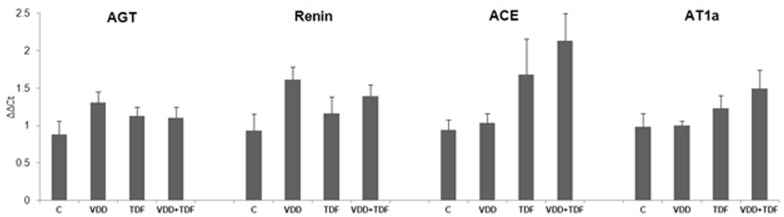
Bar graph representation of the renal mRNA expression of components of renin-angiotensin system in C (n = 5), VDD (n = 6), TDF (n = 5) and VDD+TDF (n = 6).

The renal expression of renin-angiotensin system components assessed by qPCR are given in Figure 5. AGT, renin, ACE and AT1a exhibited a tendency for upregulation in all experimental groups compared to control. As can be seen in Table 2, plasma aldosterone increased significantly and progressively in VDD, TDF and VDD+TDF groups compared to control animals. In addition, the U_K_/U_Na_ ratio was significantly higher in VDD+TDF in agreement with the more elevated aldosterona level in this group (Table 2). Altogether, this data confirms a possible involvement of the RAAS in the increase of MAP in vitamin D deficient rats treated with TDF.

**Figure 6:**
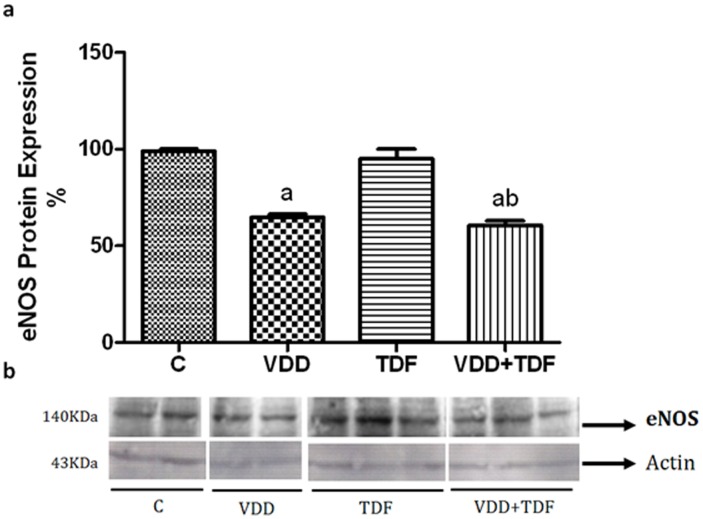
Semiquantitative immunoblotting of kidney fractions. (a) A densitometric analysis of samples from control (n = 4), VDD (n = 6), TDF (n = 6) and VDD+TDF (n = 6) rats is shown. (b) Immunoblots reacted with anti-eNOS revealing a 140-kDa band. Values are means ± SEM. ^a^p<0.001 vs. C; ^b^p<0.001 vs. TDF.

The e-NOS expression (Figure 6) was downregulated in VDD groups (VDD = 65±2%; VDD+TDF = 60±2%) compared with control (99±1%) and TDF rats (95±5%). These findings may explain the more elevated blood pressure, the intense renal vasoconstriction, and the decrease in glomerular filtration rate in VDD+TDF, and it may be an additional mechanism responsible for the increased TDF toxicity induced by vitamin D deficiency.

Serum calcium and phosphate concentrations were lower in groups that were fed a vitamin D-free diet. Decreased serum calcium and phosphate levels were also expected since the diet composition has lower calcium and phosphorus concentration (0.4% Ca and 0.4% P) compared to the standard diet. However, group TDF showed diminished serum phosphate concentration, indicating that treatment with TDF alone leads to hypophosphatemia. Association of treatment with TDF and vitamin D deficiency did not aggravate serum phosphate concentration (Table 2).

**Figure 7:**
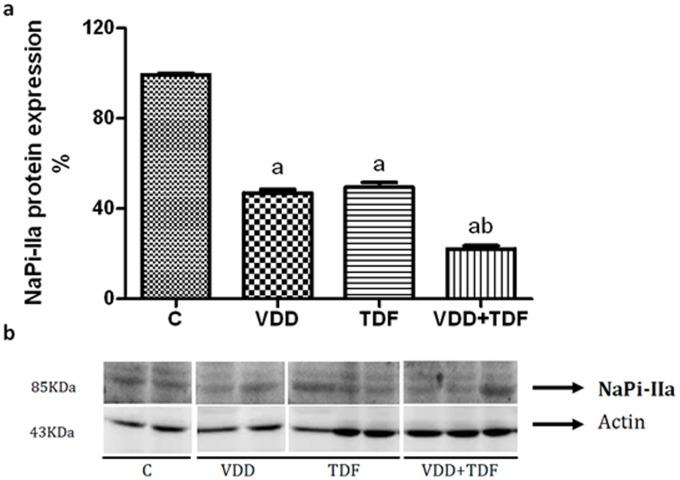
Semiquantitative immunoblotting of kidney fractions. (a) A densitometric analysis of samples from control (n = 4), VDD (n = 6), TDF (n = 6) and VDD+TDF (n = 6) rats is shown. (b) Immunoblots reacted with anti-NaPi-IIa revealing a 85-kDa band. Values are means ± SEM. ^a^p<0.001 vs. C; ^b^p<0.001 vs. TDF.

Proximal tubule function was impaired in TDF rats, as was evidenced by increased urinary excretion of phosphorus (Table 2). Association of vitamin D deficiency and treatment with TDF showed a greater impairment of proximal tubule function. To support this data, renal protein expression of NaPi-IIa was decreased by approximately 50% in VDD and TDF rats and 78% in VDD+TDF animals. Renal protein expression of NaPi-IIa is shown in Figure 7.

VDD rats showed an increased plasma PTH concentration compared to control. TDF group did not exhibit alteration in PTH level. Combination of vitamin D deficiency and TDF increased PTH compared to groups C and TDF, but this elevation was moderate compared to VDD (Table 2).

As illustrated in Table 2, TDF animals showed higher levels of total cholesterol and triglycerides compared to control group. When compared with control group, VDD animals showed higher levels of total cholesterol and triglycerides, although the latter parameter was not statistically significant. Association of vitamin D deficiency to treatment with TDF aggravated levels of both total cholesterol and triglycerides compared to groups C, VDD and TDF.

**Figure 8:**
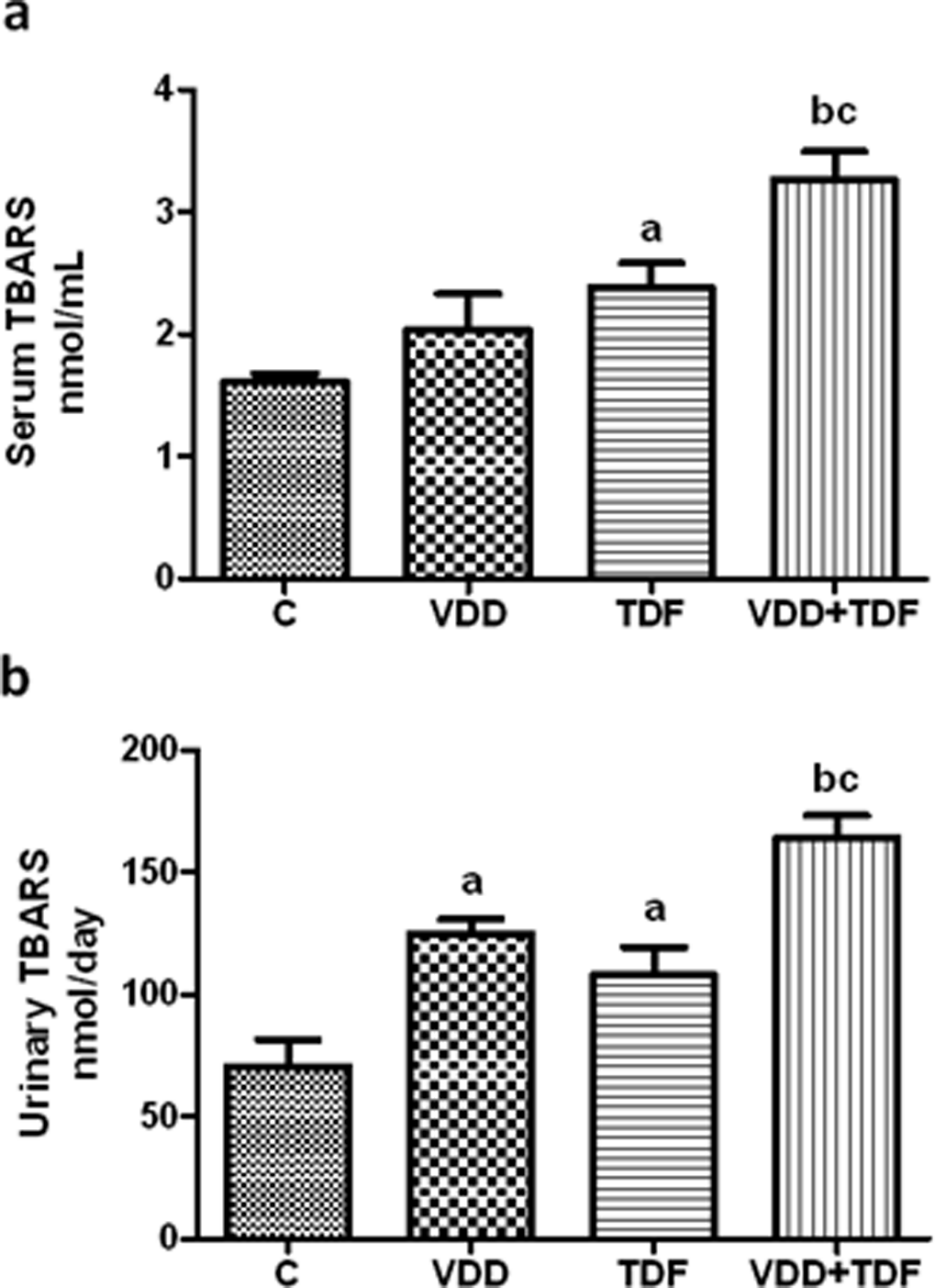
Serum and urinary TBARS. (a) Serum TBARS concentration of C, VDD, TDF and VDD+TDF groups and (b) urinary TBARS excretion of C, VDD, TDF and VDD+TDF groups. Values are means ± SEM. ^a^p<0.05, ^b^p<0.001 vs. C; ^c^p<0.01 vs. TDF.

The oxidative stress was initially evaluated by TBARS plasma concentration and urinary excretion measurements. The results are presented in Figures 8a and b. Plasma TBARS concentration (2.38±0.20 nmol/mL) and urinary TBARS excretion (107±11 nmol/24 h) was higher in group TDF when compared with control rats (serum TBARS = 1.61±0.07; urinary TBARS excretion = 70±11 nmol/24 h, p<0.05). VDD rats also exhibited a slight but not statistically significant increase in serum TBARS concentration (2.04±0.29 nmol/mL) and a higher urinary TBARS excretion (125±6 nmol/24 h, p<0.05) compared to control group probably due to the effects of vitamin D in the modulation of the oxidative stress mechanism. VDD+TDF rats showed an even more pronounced increase of serum TBARS concentration (3.26±0.23 nmol/mL) and urinary TBARS excretion (164±9 nmol/24 h).

**Figure 9:**
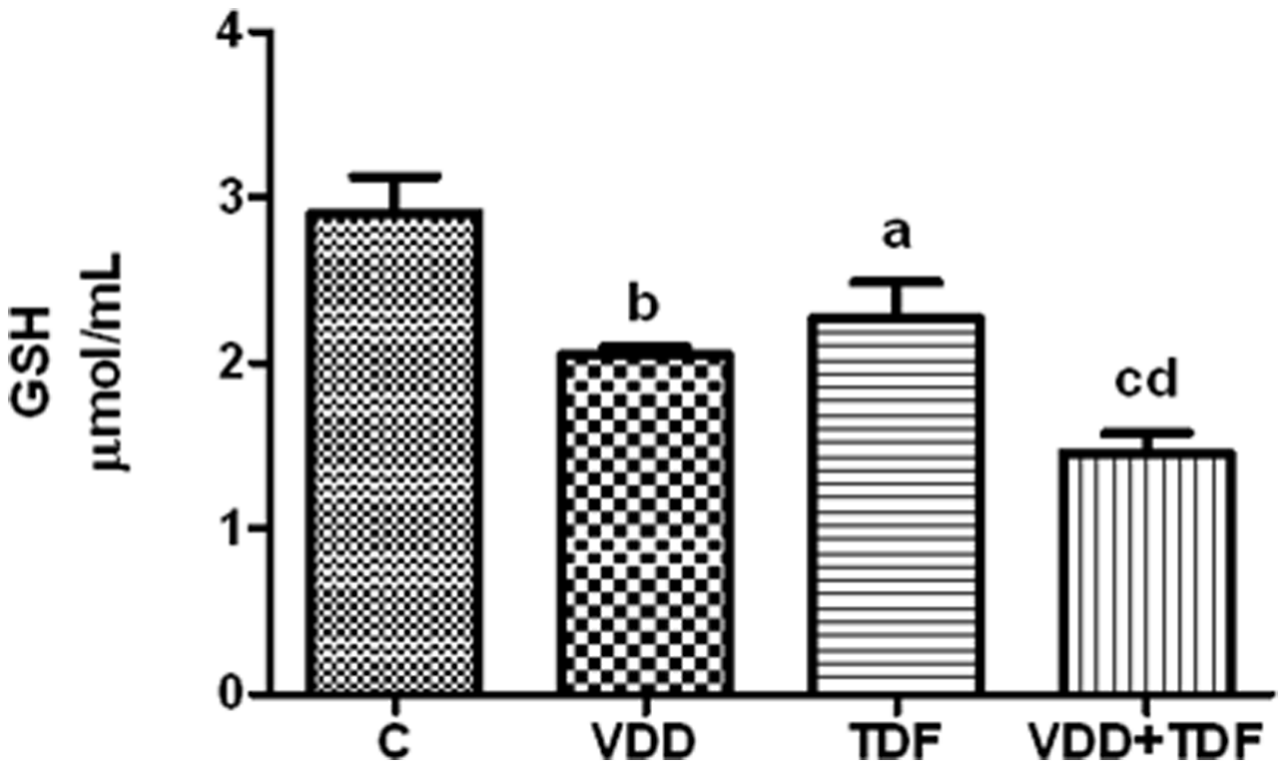
Whole blood glutathione concentration of C, VDD, TDF and VDD+TDF groups. Values are means ± SEM. ^a^p<0.05, ^b^p<0.01, ^c^p<0.001 vs. C; ^d^p<0.01 vs. TDF.

Glutathione is a major intracellular antioxidant agent. As shown in Figure 9, the total gluthatione concentration in control rats was 2.90±0.22 µml/mL, a value statistically higher than VDD group (2.05±0.04 µml/mL, p<0.01) and TDF (2.27±0.22 µmol/mL, p<0.05). VDD+TDF presented markedly lower glutathione concentration compared to groups C, VDD and TDF (1.46±0.12 µml/mL).

## Discussion

Vitamin D deficiency or insufficiency has been increasing in recent years reaching 73% of total HIV-infected patients and has been associated with HIV disease progression and its related complications [7], [8], [22]. Treatment with TDF led to renal failure, proximal tubule injury, hypertension, abnormal lipid metabolism and oxidative stress. Our data demonstrated that vitamin D deficiency aggravates TDF-induced nephrotoxicity.

Our data showed that circulating vitamin D levels were undetectable in VDD and VDD+TDF groups (<1.5 ng/mL), while C and TDF groups presented 15.4±1.0 and 14.8±1.3 ng/mL respectively. Thus, our vitamin D deficiency model was successfully developed. Previous studies demonstrated that basal levels of vitamin D in rats range from 12 to 20 ng/mL [23], [24]. Moreover, Ismail et al [25] using a 6-week-vitamin D deficiency model in Wistar rats showed that the levels of 25(OH)D were approximately 1.0 ng/mL in vitamin D-deficient animals while control group presented around 20.0 ng/mL.

*Scherzer et al* [26] showed that TDF use was associated with proteinuria and chronic kidney disease. Furthermore, vitamin D deficiency itself has been associated with increased prevalence of proteinuria in adults. Vitamin D is a known negative endocrine regulator of the RAAS, which plays a key role in the development of kidney disease. *Li et al* [27] demonstrated that VDR-null mice presented higher renin and angiotensin II levels leading to hypertension. Moreover, low levels of vitamin D also induce podocyte loss and the development of glomerulosclerosis through direct cellular effects, compromising the integrity of the glomerular filtration membrane [10], [28].

### Hypertension, renin-angiotensin-aldosterone system and nitric oxide in TDF-induced nephrotoxicity associated with vitamin D deficiency

In this study, vitamin D deficient rats receiving TDF showed higher blood pressure. This alteration was accompanied by a marked increase in protein expression of angiotensinogen and AT1 receptor. In addition, gene expression of some RAS compounds such as renin, AGT, ACE and AT1a were also augmented. Furthermore, we demonstrated a progressive elevation in plasma aldosterone in VDD, TDF and VDD+TDF groups. Previous studies have shown strong evidences that vitamin D deficiency led to an upregulation of the renin-angiotensin system [29], [30]. Studies conducted in humans and animals reported that gene expression and plasma renin activity are increased in vitamin D-deficient patients [31] and vitamin D receptor knockout mice [32]. Since our findings also revealed increased activation of RAAS, it is possible to speculate that the more severe hypertension observed in VDD+TDF could be explained by the increased mRNA expression of renin, AGT, ACE, and AT1a associated with augmented levels of aldosterone. In addition, our data showed higher expression of angiotensin II and its receptor in the kidney of VDD+TDF animals, reinforcing the role of vitamin D on blood pressure control. Moreover, it is important to point out that vitamin D deficient rats treated with TDF exhibited a higher urinary concentration of potassium and sodium ratio suggesting an increased potassium secretion in the distal tubule due to more elevated levels of aldosterone [33].

It is well known that oxidative stress is also implicated in the development of hypertension. Angiotensin II activates NADPH oxidase leading to the generation of superoxides [34], [35]. Our data demonstrated that vitamin D deficient rats treated with TDF showed hypertension accompanied by elevated RVR and decreased renal eNOS expression, suggesting the possibility of the involvement of nitric oxide (NO) cascade. Within the kidney, e-NOS is the main NOS isoform responsible for NO production in both renal epithelial and endothelial cells. Nitric oxide regulates blood pressure by its effects on vascular tone, renal haemodynamics, sodium balance and extracellular fluid volume. Kopkan el al [36] reported that mice lacking the gene for eNOS developed salt-sensitive hypertension due to oxidative imbalance to keep the optimal production of NO. The decrease in eNOS expression in vitamin D deficient rats treated with TDF may explain the more severe hypertension observed in this group.

### The role of PTH and vitamin D deficiency in calcium and phosphorus metabolism associated with TDF

Our study showed that vitamin D deficient rats presented hypophosphatemia, hypocalcemia and higher levels of PTH. These alterations were expected since the lack of vitamin D reduces calcium intestinal absorption leading to a lower level of calcium and higher production of PTH by the parathyroid gland. PTH, in turn, acts on bone tissue in order to attenuate the decrease in serum calcium and the increase in phosphorus excretion.

Treatment with TDF led to proximal tubule injury, evidenced by higher excretion of phosphorus and decreased renal expression of the NaPi-IIa cotransporter leading to hyperphosphaturia and hypophosphatemia. Combination of vitamin D deficiency and treatment with TDF aggravated these tubular alterations. Kurnik *et al* reported that treatment with 1,25(OH)_2_D_3_ decreased urinary phosphate excretion in partially vitamin D depleted rats, indicating a physiological stimulatory role of 1,25(OH)_2_D_3_ in renal phosphate transport [37].

Vitamin D deficient rats presented high levels of plasma PTH supporting previous studies that reported a relationship between the highest PTH levels and the lowest vitamin D concentrations [38]. It is well known that vitamin D and calcium are potent regulators of PTH, suggesting that hypocalcemia and hypovitaminosis D combined with a slight decrease in renal function, as observed in VDD animals, may be responsible for the increase in PTH. This may occur in order to restore these parameters in CKD. The hyperphosphaturic effect of TDF may explain the lower levels of PTH in VDD+TDF when compared with VDD group.

### TDF and vitamin D deficiency lead to metabolic syndrome

TDF rats showed higher levels of triglycerides and total cholesterol. These parameters were aggravated when TDF was administered in association with vitamin D deficiency. *Gagnon et al* [17] demonstrated that vitamin D levels were associated inversely with the risk of developing metabolic syndrome. To support data previously shown, another study reported that mice with type 1 and 2 *diabetes mellitus* treated with doxercalciferol showed a higher renal VDR renal expression and the activation of this receptor led to a reduction in the accumulation of triglycerides and cholesterol. Moreover, dyslipidaemia is a frequent complication among HIV-infected patients on antiretroviral therapy [39], [40].

### Oxidative stress is associated with renal injury and renovascular alterations

The association of vitamin D deficiency and treatment with TDF led to the increase of lipid peroxidation and the decrease in the major intracellular antioxidant agent, observed by higher TBARS and lower GSH levels respectively, showing the influence of oxidative stress in the development of renal injury and the onset of hypertension. *Borges-Santos et al* [15] reported that HIV-infected individuals presented an antioxidant deficit, evidenced by higher levels of oxidized glutathione (GSSG), contributing to disease progression. Furthermore, there are several studies regarding the indirect effects of vitamin D on the protection against oxidative stress. *Tarcin et al* [14] showed a negative correlation between 25(OH)D_3_ levels and TBARS in vitamin D deficient patients and the protective effect of vitamin D on endothelial function. Experimental studies reported that treatment with calcitriol led to an increase of VDR expression and decrease in malondialdehyde levels, supporting the role of vitamin D in protecting DNA against oxidative damage [41].

### Final considerations

In summary, our data confirms that vitamin D deficiency aggravates TDF nephrotoxicity at least in part by the increase of oxidative stress and the involvement of renin-angiotensin system and nitric oxide cascade, demonstrating that vitamin D has an essential role in the development and progression of kidney and cardiovascular diseases in HIV-infected subjects. Hence, it is important to monitor vitamin D levels in HIV-infected patients treated with TDF. Further studies are required for the clarification of specific mechanisms involved in the progression of HIV-related diseases in vitamin D deficient individuals.
